# Enhancing the Depth of Analyses with Next-Generation Ion Mobility Experiments

**DOI:** 10.1146/annurev-anchem-091522-031329

**Published:** 2023-03-31

**Authors:** Benjamin P. Zercher, Theresa A. Gozzo, AnneClaire Wageman, Matthew F. Bush

**Affiliations:** Department of Chemistry, University of Washington, Seattle, Washington, USA

**Keywords:** ion mobility, mass spectrometry, multidimensional analysis, biomolecules, molecular identification, structural elucidation

## Abstract

Recent developments in ion mobility (IM) technology have expanded the capability to separate and characterize gas-phase ions of biomolecules, especially when paired with mass spectrometry. This next generation of IM technology has been ushered in by creative innovation focused on both instrument architectures and how electric fields are applied. In this review, we focus on the application of high-resolution and multidimensional IM to biomolecular analyses, encompassing the fields of glycomics, lipidomics, peptidomics, and proteomics. We highlight selected research that demonstrates the application of the new IM toolkit to challenging biomolecular systems. Through our review of recently published literature, we outline the current strengths of respective technologies and perspectives for future applications.

## INTRODUCTION

Ion mobility spectrometry, or simply ion mobility (IM), was introduced more than 120 years ago (1) and now boasts a flourishing partnership with its younger sibling, mass spectrometry (MS). IM separates gaseous ions in the presence of an electric field, E, and a buffer gas; the steady-state velocity of the ion, $v_{\text{D}}$, is proportional to E according to the mobility of the ion, K:

1. $$K=\frac{v_{\text{D}}}{E}=\frac{L}{t_{\text{D}}E}.$$

K can be determined from the ion’s drift time, $t_{\text{D}}$, and the length, L, of the separation region. Note that $t_{\text{D}}$ refers to the residence time of the ion in the separation region; researchers often report the arrival time ($t_{\text{A}}$) of ions at a detector that is located distal to the separation region (2). Within the low-field limit, the ion-neutral collision cross section, Ω, can be determined from K using the Mason-Schamp equation (3, 4):

2. $$\text{Ω}=\frac{3ez}{16N}{\left(\frac{2\pi }{\mu k_{B}T}\right)}^{1/2}\frac{1}{K},$$

where e is the elementary charge, z is the charge state, N is the drift-gas number density, μ is the reduced mass of the ion-drift-gas pair, $k_{B}$ is the Boltzmann constant, and T is drift-gas temperature. Overviews of the fundamentals of IM are available elsewhere (2, 5). IM continues to be used widely as a stand-alone technique (6–8). Technology for IM-MS has advanced rapidly in recent years, as highlighted in several reviews (2, 9–12).

**Figure 1:**
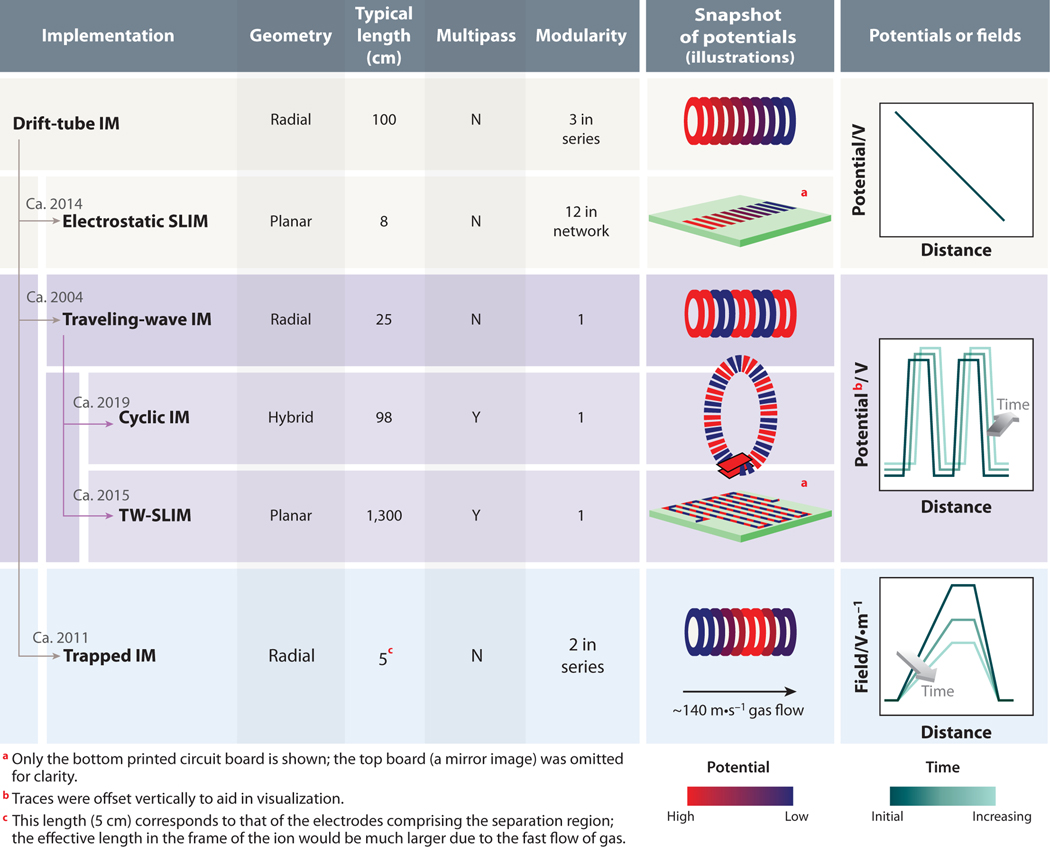
Summary of selected implementations of ion mobility (IM), including drift-tube IM, electrostatic structures for lossless ion manipulations (SLIM) (30), traveling-wave IM (27), cyclic IM (32), traveling-wave (TW) SLIM (31), and trapped IM (129). To enable higher-performance experiments, technologies that have followed on from drift tubes have applied innovations in applied electric fields, electrode geometries, the ability to subject ions to multiple passes through the separator, and increased modularity/dimensionality. The years adjacent to the arrows correspond to the publications cited above and do not consider earlier disclosures in patents or conference presentations.

Today, concerted research efforts have pushed IM to the precipice of a new generation of technology, as IM separations now access unprecedented performance and experimental flexibility (13). To understand today’s instrumentation, we provide a summary of selected IM technologies in Figure 1. In drift-tube IM, ions experience a uniform electrostatic field established by potentials created by a voltage divider network that are applied to a series of ring electrodes (14) (Figure 1). Using drift-tube IM, Clemmer, Jarrold, Bowers, and Hill, among others, made pioneering contributions to our understanding of the structures of peptide and protein ions from single-component samples (14–20). The ability of IM to separate similar structures depends on the resolving power, $R_{p}$, of the measurement, which exhibits the following relationship for drift-tube IM (3):

3. $$R_{p}=\frac{t_{\text{D}}}{t_{FWHM}}∼{\left(\frac{LE}{T}\right)}^{1/2},$$

where $t_{FWHM}$ is the full width at half maximum (also used below as fwhm) of the feature in drift time. Zimmermann and coworkers (21) have reviewed implementations that optimize $R_{p}$. Improving the $R_{p}$ of drift tubes can create additional challenges. For example, increasing L at constant E is associated with greater radial diffusion and decreased transmission, which has since been addressed using electrostatic focusing (22, 23) and radio frequency confinement (24). Increasing L also requires larger instrument footprints and the application of higher potentials due to the relationship between L and E. A complementary way to increase the information content of IM measurements is to increase dimensionality. Clemmer and coworkers (25, 26) pioneered IM-IM-MS, or tandem IM, by interfacing a series of drift tubes using mobility-selective ion funnels.

Electrodynamic separations enable alternative strategies to increase the performance of IM measurements. In traveling-wave IM, a series of potentials is propagated down the length of an IM cell as a function of time (Figure 1). Individual waves push ions down the length of the device until the drag from gas collisions forces ions to roll over the wave. The propensity of ions to experience roll-over events decreases with increasing K, thus enabling mobility-dependent separations (27). In contrast to drift-tube IM, absolute voltages depend on the amplitude of the traveling wave, rather than L. Another electrodynamic strategy is trapped IM spectrometry (TIMS), which utilizes a directional flow of gas to propel ions and a countering E gradient that impedes their progress (28) (Figure 1). Under those conditions, ions reach an equilibrium position that depends on K. As the gradient is relaxed, ions are eluted in order of decreasing K (29). The performance of the separation depends strongly on the rate of gradient change, i.e., the scanning speed; TIMS can achieve high-performance $R_{p}$ without lengthening the physical length of the cell (28).

Drift-tube, traveling-wave, and trapped IM were all first implemented using cells with a radial axis of symmetry (Figure 1). In addition to electrodynamic fields, recent IM devices have also benefited from alternative geometries. Structures for lossless ion manipulations (SLIM) introduced a planar geometry using mated pairs of printed circuit boards; implementations have utilized both electrostatic (30) and traveling-wave (31) separations (Figure 1). The recently introduced cyclic IM (cIM) system makes innovative use of elements with planar and radial geometries (32). The combination of new architectures and electrodynamic strategies forms the basis of next-generation IM instrumentation, pushing the boundaries of separation performance for biomolecular applications.

IM performance is often reported using single-peak $R_{p}$:

4. $$R_{p}=\frac{x}{\text{Δ}x},$$

where x is the peak centroid, and Δx is the width of the peak, usually defined as the fwhm. Traditionally, the independent variable of the experiment, drift time, has been used to define $R_{p}$ in IM, i.e., $R_{p}^{t}$ (33). However, the broader adoption of electrodynamic separations has complicated the evaluation of this figure of merit. For one, electrostatic separations exhibit a linear relationship between $t_{\text{D}}$ and $K^{-1}$, whereas electrodynamic separations exhibit a nonlinear relationship between those quantities. Many researchers report resolving powers in the Ω space, i.e., ${R_{p}}^{\text{Ω}}$; this requires converting $t_{\text{A}}$ to Ω using a calibration function (34). The nonlinear relationship between $t_{\text{D}}$ and Ω in a calibration function results in different values for ${R_{p}}^{t}$ and ${R_{p}}^{\text{Ω}}$ (34). Additionally, ${R_{p}}^{t}$ is not descriptive of IM separations that utilize electric field scanning, such as TIMS. Alternatively, the peak-to-peak resolution, $R_{p-p}$, has also been used to describe the separation of two analytes in IM (31, 35):

5. $$R_{p-p}=2\left|\frac{x_{2}-x_{1}}{\text{Δ}x_{2}+\text{Δ}x_{1}}\right|,$$

where $x_{2}$ and $x_{1}$ are the centroid values, and $\text{Δ}x_{2}$ and $\text{Δ}x_{1}$ are the widths, often fwhm, of the two peaks, usually in terms of arrival time. As next-generation instrumentation accesses unforeseen separation capabilities, one goal of this review is to provide clarity in the methods used to report resolution for different implementations of IM.

This review focuses on the applications of next-generation IM instrumentation to biomolecular analysis. It complements recent reviews of fundamentals of the various IM techniques (5, 12, 28, 36, 37) and multidimensional IM separations (13). We are motivated by the recent research thrust and commercialization of IM instruments that redefine IM paradigms and access ultrahigh-resolution separations or multidimensional experiments. Specifically, we focus on SLIM, cIM, and TIMS as applied to a range of applications including glycomics, peptidomics, lipidomics, and proteomics. Additional aspects of the separation fundamentals and figures of merit of these techniques have been reviewed elsewhere (21). These techniques all disperse ions in time based on mobility (11). Techniques that filter based on mobility, e.g., field asymmetric waveform ion mobility spectrometry (FAIMS, also referred to as differential mobility spectrometry) (38), overtone mobility spectrometry (39, 40), or differential mobility analysis (41, 42), are not included in this review.

## STRUCTURES FOR LOSSLESS ION MANIPULATIONS

SLIM uses mated pairs of printed circuit boards (30), which are more amenable to large, complex designs and rapid prototyping than ring electrode approaches that require extensive fabrication and soldering. The initial implementations of SLIM used electrostatic potentials to separate ions (30). The voltage gradient in electrostatic SLIM was designed to mimic that of a drift tube, but with planar symmetry. Early simulation-based work characterized the ion dynamics and electric potentials resultant from the interaction of electrostatic fields and superimposed radio frequency potentials used for ion confinement (43, 44). Experimental work then used time-dependent potentials for mobility-resolved ion selection (45) and ion trapping (46, 47) on electrostatic SLIM. These initial studies primarily analyzed relatively small molecular ions, e.g., 500–1,000 m/z (46, 47), but additional work used these strategies to determine Ω values of native-like protein ions with masses up to 145 kDa (48). Follow-on studies have included the use of series and networks of modular electrostatic SLIM devices to enable tandem IM (49), including time-dependent studies of native-like protein ions (50).

**Figure 2:**
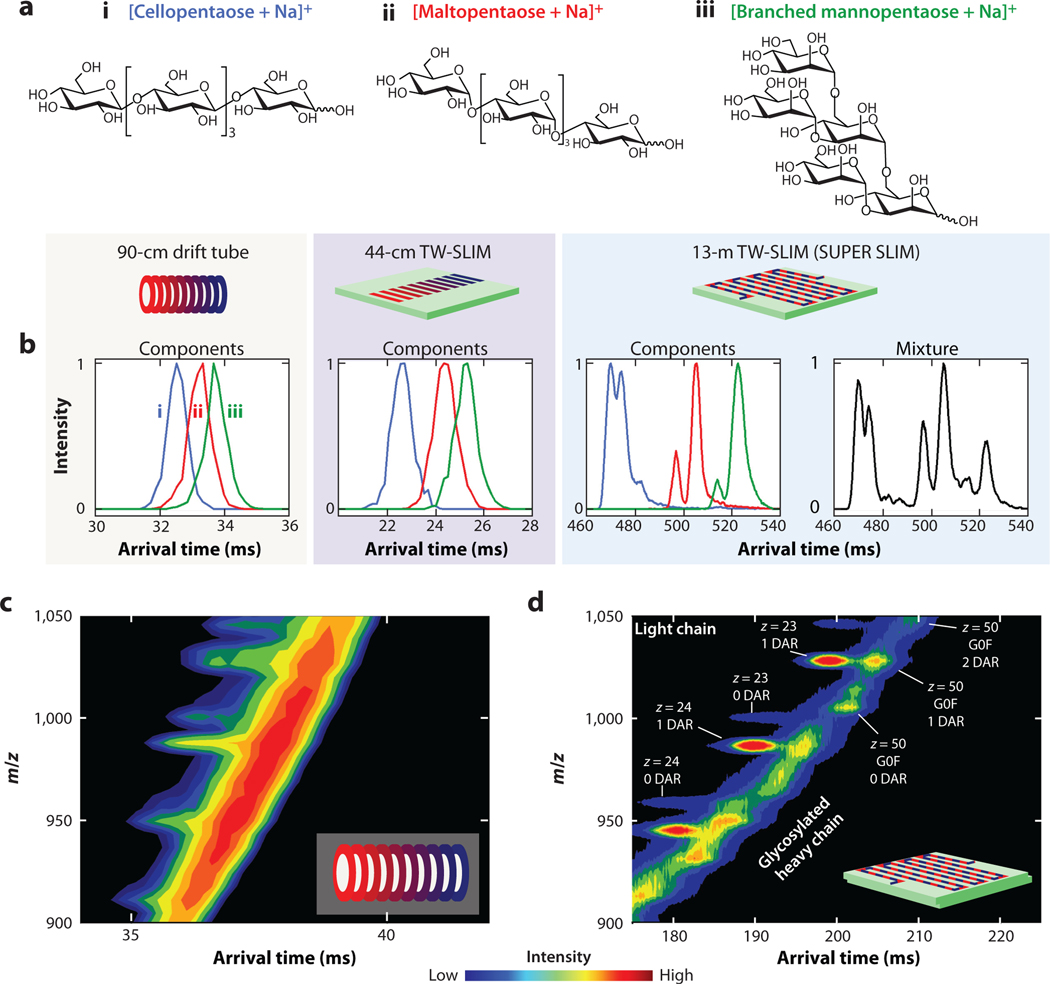
(*a*) Structures of selected isomeric pentasaccharides. The graphics (reproduced from Figure 1) indicate the IM implementation used to measure the (*b*) arrival-time distributions of selected pentasaccharide ions. From left to right, the distributions were measured using a 90-cm drift tube, 44-cm TW-SLIM device, 13-m TW-SLIM device, and the same 13-m TW-SLIM device, respectively. Panel *b* adapted with permission from Reference 55; copyright 2016 John Wiley & Sons. IMS-MS heatmap of a reduced antibody-drug conjugate on a commercial 1-m drift-tube IM system (*c*) and a 4.5-m TW-SLIM system (*d*). Panels *c* and *d* adapted with permission from Reference 56; copyright 2020 American Chemical Society. Minor modifications were made to the heatmap in panel *d* to clarify labeling. Abbreviations: DAR, drug:antibody ratio; G0F, denotes glycosylation with N-linked glycan; IM, ion mobility; MS, mass spectrometry; SUPER, serpentine ultralong path with extended routing; TW-SLIM, traveling-wave structures for lossless ion manipulations.

Traveling-wave IM has also been implemented using the SLIM architecture (TW-SLIM) (31). Upon optimization of the traveling-wave profile, the TW-SLIM module maintained lossless transmission while exhibiting an ${R_{p}}^{t}$ of 27.0 for the 622 m/z hexakis phosphazene ion of a commercially available tuning mix (31). Early studies also reported observations of ion surfing (27), wherein ions with sufficiently large K are transported between two waves at their velocity. Although no separation occurs, narrow peaks result in erroneously high $R_{p}$ values, as even the effects of longitudinal diffusion are limited (31).Subsequently, separation quality was maintained on a TW-SLIM module that incorporated 90° turns (51).This enabled the creation of long, compact, serpentine pathlengths; a SLIM module with a 13-m pathlength was introduced that exhibited fivefold-higher resolution compared to commercially available traveling-wave and drift-tube IM systems at that time (52). To further decouple the pathlength from the overall size of the IM region, an ensuing modification enabled the recycling of ions for multiple passes around the 13-m device, referred to as serpentine ultralong path with extended routing (SUPER) SLIM (53). The initial study reported peak-to-peak resolution for hexakis phosphazene ions of m/z 622 and 922; after 40 passes on SUPER SLIM,this yielded a 30-fold-higher resolution compared to a commercially available drift tube (53). Early applications of SUPER SLIM have been reviewed elsewhere (54). The improvement of resolution afforded by longer separations using TW-SLIM devices is demonstrated in the comparison of different instrumental platforms in Figure 2. In Figure 2b, isomeric pentasaccharides are separated on the SUPER SLIM system, revealing further conformational heterogeneity attributed to potential anomerism and sodium protonation sites that was unresolved on other platforms (55). Additional recent work used SUPER SLIM to characterize drug loads of the heavy and light chains of a monoclonal antibody (mAb) (56). Figure 2c,d shows a comparison between data obtained using drift-tube IM and SUPER SLIM. Relative to drift-tube IM, SUPER SLIM separations better resolved the drug loads of both the light and heavy chains, potentially increasing analytical throughput relative to traditional liquid chromatography (LC) separations.

One challenge of SLIM and other IM techniques with extremely long pathlengths is that ions experience significant diffusion along the axis of separation; this results in low fluxes of ions that can hinder some experiments. Compression ratio ion mobility programming (CRIMP) was used to compress ions dispersed in time and space by utilizing a traditional traveling wave followed by a stuttering traveling wave (57). This strategy was used in a 99-m separation that resolved peptide epimers of β-amyloid using SUPER SLIM (58). Although the separation pathlength for recycled ions in SUPER SLIM is theoretically unlimited, the IM resolution is limited by the wrap-around effect, wherein higher K analytes begin to overtake lower K analytes. To address this, a multilevel SLIM device was constructed; ions were transported to different levels of the device using ion escalators, accessing a separation pathlength of 43.2 m and ${R_{p}}^{\text{Ω}}$ of 560 (59). This system was then miniaturized such that the multilevel device had a pathlength of 1 m and achieved an ${R_{p}}^{\text{Ω}}$ of 131, which is 1.5 times higher than a 78-cm drift-tube IM system (60).

MOBILion Systems recently introduced a system with a 13-m TW-SLIM module aimed at providing both high-resolution and high-throughput separations. On this instrument, isomeric and isobaric compounds that could not be separated by MS alone were separated and exhibited an ${R_{p}}^{\text{Ω}}$ more than 200 (61). TW parameters were optimized in terms of $R_{p}$ while limiting ion surfing; these wave conditions also resulted in the lowest errors in the resulting calibrated Ω values (61). This commercialized instrument has been used to separate gangliosides without additional LC separation, achieving baseline resolution of two isomers that differed only in the location of a single sialic acid linkage (62). This IM-MS workflow without LC represented a 15-fold improvement in throughput compared to traditional LC separations, addressing an analytical bottleneck in shotgun lipidomics (62). This system has also been coupled with LC to quantify post-translational modifications of pharmaceutically relevant peptide therapeutics (63). Specifically, the addition of the IM dimension enabled the separation of coeluting isomerized aspartic acid-containing peptides as well as a deamidation from a parent mAb peptide, both of which were unresolved by LC (63). As the resolution and runtime of LC separations are inversely related, this study illustrates the potential utility of high-resolution IM to reduce runtimes for complex separations.

SLIM technologies have also been developed for specific application to glycans, whose characteristics of branching, anomerism, and heterogeneity pose challenges to traditional analysis methods. Rizzo and coworkers (64) have combined TW-SLIM with cryogenic infrared action spectroscopy (cryo-IR) to differentiate and identify glycans. First, a 1.8-m TW-SLIM device with ion recycling capability coupled with a combination cryogenic ion trap/time-of-flight mass spectrometer was used to separate and identify individual components of a mixture of epimers as well as a set of two tetrasaccharide isomers found in human milk. α-_D_-glucose was mobility separated at different time points to monitor the anomeric conversion to β-_D_-glucose in real time according to the cryo-IR profiles, enabling the calculation of rate constants $k_{\text{α}}$ and $k_{\text{β}}$ in agreement with literature values (65). Additionally, a spectral decomposition algorithm was developed to identify component glycans from mixtures, with potential for application to lower-resolution mobility separations (66).

Further instrumental innovations sought to increase the throughput and performance of the IM/cryo-IR workflow. A new instrument was introduced that incorporated a SLIM module with a 2-m on-board accumulation region that increased ion utilization efficiency, followed by a 10-m multipass TW-SLIM device that separated GRGDS and SDGRG ions at ${R_{p}}^{\text{Ω}}$ values of up to ~1,000 (67). Furthermore, principal component analysis was used to decrease the wavenumber range required for fingerprinting, reducing IR acquisitions to as little as 5 s (67). This instrument was then used to separate, characterize, and identify positional isomers of an N-linked glycan associated with the crystallizable region of immunoglobulins (IgGs) in a 60-m separation (68).The two closest isomeric peaks in the arrival-time distribution were estimated to differ by 0.2–0.3% in terms of Ω which is below the uncertainty of Ω values and precludes a database approach using Ω alone (68). By adding orthogonality to the ultrahigh-resolution IM separation, the partnership of SLIM and cryo-IR offers exciting potential for glycan characterization.

**Figure 3:**
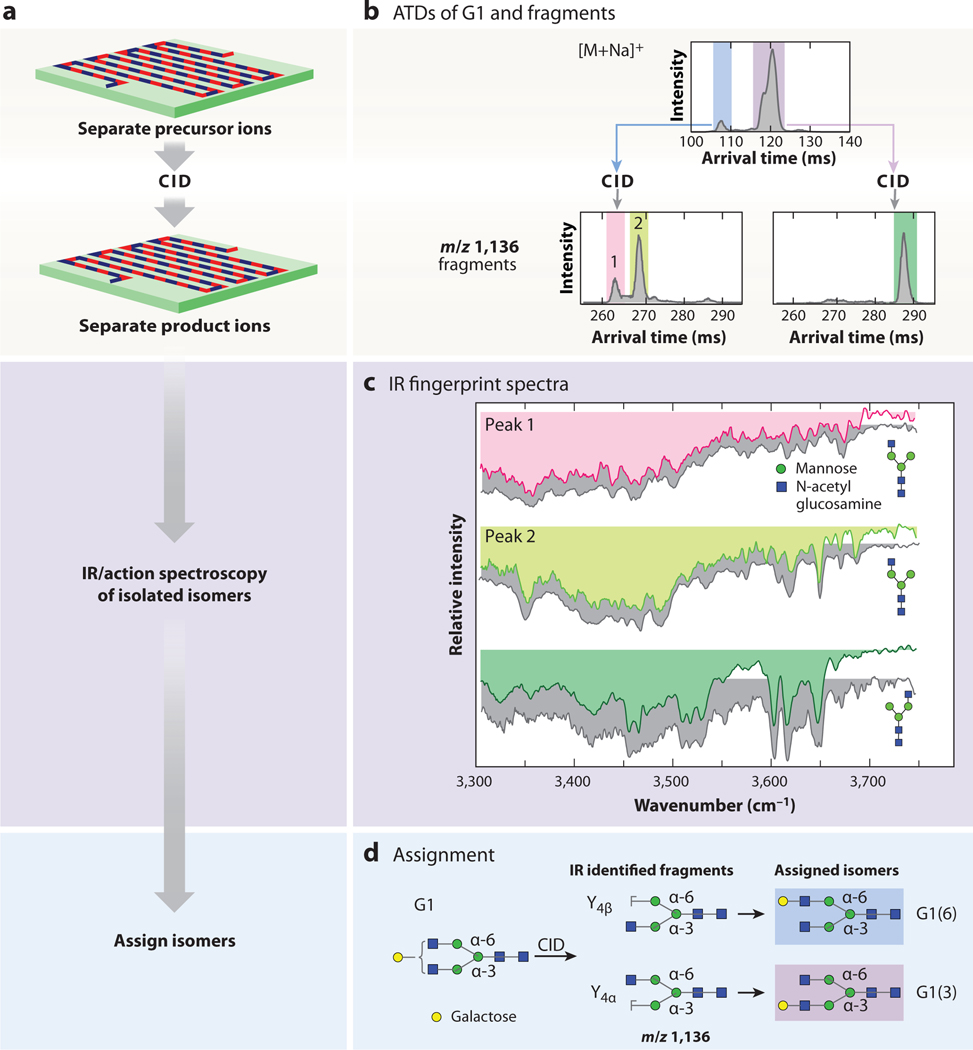
(*a*) Schematic representation of the IM/CID/cryo-IR workflow using the graphics from Figure 1. (*b*) ATDs of precursor G1 separated on a TW-SLIM device with a 10-m pathlength (*top*); peaks highlighted in blue and purple underwent CID, and m/z 1,136 fragments underwent additional IM separation (*bottom*). (*c*) IR fingerprint spectra of mobility-separated m/z 1,136 fragments were recorded and matched to database spectra (*gray*) of fragments from G0-N. Fragment IR spectra are color coded to match mobility peaks from the ATDs for fragments in panel *b*. (*d*) Structures of the m/z 1,136 fragments were assigned based on reference spectrum comparison. Isomer structures are highlighted in blue and purple to match mobility peaks from the G1 ATDs in panel *b*. Panels *b*, *c*, and *d* adapted with permission from Reference 70; copyright 2022 Royal Society of Chemistry. Abbreviations: ATD, arrival-time distribution; CID, collision-induced dissociation; IM, ion mobility; IR, infrared; TW-SLIM, traveling-wave structures for lossless ion manipulations.

The latest version of this platform also includes on-board trapping regions capable of collision-induced dissociation (CID) (69). Following CID, ions can be either recycled for additional IM separation or directly analyzed by cryo-IR or MS (69). For example, IR spectra of the C-fragment of the human milk oligosaccharide LNnT were compared with those measured for disaccharide standards, which indicated that the anomeric configuration of the glycosidic bond in the C-fragment was preserved during fragmentation (69). This finding and others illustrate the effectiveness of the database approach: Determining the structures of small intact glycans and glycan fragments by comparison to available standards enables the structural elucidation of larger and more complex glycans that usually lack appropriate standards (70–72). For example, Figure 3 illustrates the application of this strategy to N-glycans G0-N and G1 (70). IR spectra of selected Y-fragments of G0-N were compared to relevant standards to determine isomeric identity (70). The IR spectra of the G0-N fragments were then used to identify fragments of the G1 glycan produced by post-IM CID (70) (Figure 3). The same approach was used to assign anomericity of larger glycans LNnT and maltopentaose by comparing the IR spectra of selected fragments to those of anomerically pure corresponding mass analytical standards (71). Adding CID to the IM/cryo-IR workflow has enabled bottom-up sequencing and isomer identification of increasingly large glycans with more isomeric possibilities that were previously undifferentiable.

## CYCLIC ION MOBILITY

The cIM system was introduced by Waters Corp. and incorporates many innovations for next-generation IM experiments (32). Whereas most IM separations occur along the primary axis of the instrument, the cIM system incorporates a 98-cm, circular TW separator that is orthogonal to the primary axis. The interface of the IM separator and the primary axis consists of a pair of intersecting arrays of planar electrodes that control ion motion in that region. Ions can be subjected to an arbitrary number of passes around the cIM device to access increasingly high-resolution separations. Pre- and postarray ion stores enable mobility-based selection, storage, and activation of ions during various intervals of the experiment. The tunable resolution and flexible experimental modes overcome a traditional limitation of IM technologies, in which the geometry of the instrument imposes constraints on the pathlength and dimensionality of experiments.

The cIM system exhibits significantly improved resolution relative to previously commercialized traveling-wave IM instruments. The first- and second-generation hybrid quadrupole/ traveling-wave IM/time-of-flight systems exhibited ${R_{p}}^{\text{Ω}}$ values near 10 (73) and 45 (74), respectively. Using cIM, ions of GRGDS and SDGRG exhibited an ${R_{p}}^{\text{Ω}}$ of ~350 after 16 passes (32). As with SUPER SLIM, ion wrap-around can hinder the separation of two analytes as the higher-K ions overtake lower-K ions; mobility-based isolation was used to select SDGRG and subject it to additional passes around the cIM in the absence of GRGDS. ${R_{p}}^{\text{Ω}}$ increased with the square root of the number of passes, consistent with expectations from theory, and reached ~750 after 100 passes (32). Many subsequent studies using cIM report estimated ${R_{p}}^{\text{Ω}}$ values based on those earlier measurements for GRGDS and SDGRG (32), rather than a value based on new observations for the analytes of interest.

The cIM system has been used to analyze many samples that were challenging to resolve using earlier IM systems. Acyl glucuronides are metabolites of nonsteroidal anti-inflammatory drugs (NSAIDs); their degradation yields biosynthetic isomers that are associated with adverse reactions. Unresolved using earlier traveling-wave IM systems, acyl glucuronide isomers were resolved using 8 passes of cIM (75). Although these isomers can also be separated using high-performance LC, the cIM system enables much shorter analysis times that are amenable to high-throughput methods for highly reactive metabolites (75). The cIM system was also used to distinguish α and β anomers of monosaccharides using 5 passes (76). Chiral systems also present analytical challenges for characterization. In the analysis of a racemic thalidomide mixture, multipass cIM distinguished dimers based on their diastereomeric composition (77). Although unable to distinguish enantiomeric monomers, the ultrahigh resolving power of cIM (${R_{p}}^{\text{Ω}}>400$) enabled differentiation by a self-association mechanism, which could be leveraged in other systems to determine optical purity without the need for additional chiral modifiers (77).

Carbohydrates are the subject of a focused research effort using high-resolution cIM separations to address analytical challenges owing to their high isomeric complexity. The cIM system was used to characterize the arrival-time distributions of oligosaccharides as a function of their group I metal adducts and degrees of polymerization (78). cIM analyses ranging from 5 to 15 passes revealed that multiple features of the IM distribution arose due to α/β anomerism (78). Further cIM characterization of oligosaccharides was pursued in the development of a library linking Ω distributions and IR spectra for a fingerprinting approach to determine oligosaccharide ring size (79). Chemoenzymatically synthesized disaccharides were characterized using 4 cIM passes to reveal unique features in the Ω distributions (79). Additional studies on the structure of chemoenzymatically synthesized glycans utilized tandem LC-cIM-MS to identify 8 distinct conformations (80). In another study, cIM was used to distinguish glycopeptide fragments to characterize the O-glycosylation of the SARS-CoV-2 spike protein; 5 passes were needed to separate different sialic acid linkages (81).

In addition to the enhanced resolution provided by multipass experiments, ions can be subjected to CID in the trap cell or the transfer cell, or upon injection or reinjection into the cIM region (32, 82). CID-cIM, which refers to activation prior to cIM, can be performed in the trap cell or upon injection into the cIM region. cIM-CID, which refers to activation after cIM, can be performed in the transfer cell. Because no additional separation takes place after activation, the arrival-time distributions of any fragmented or unfolded ions align with the associated precursors (82). Finally, these approaches can be employed with tandem IM or IM^n^, e.g., cIM-CID-cIM, cIM-CID-cIM-CID, and CID-cIM-CID.

Different cIM and CID combinations reveal distinct information. In CID-cIM, fragment ions are separated in IM, and their own arrival-time distributions are recorded. This is useful when isomeric precursor ions yield similar product ion mass spectra, even when the products may have different structures. With pre-cIM fragmentation, isobaric fragments can be associated with specific drift times. For example, from the short reference peptide HLSDSR, an isomeric peptide mix was synthesized with site-specific modifications, including D and L enantiomeric amino acids (83). With an ultraperformance LC-compatible method, the peptides were separated well in retention time-drift time space; however, if the peptide modifications were not known a priori, their sites could not be identified based on their retention time and $t_{\text{D}}$ alone, or with a cIM-CID approach. With quadrupole selection, CID in the trap, and cIM separation, the shifts of the arrival-time distributions for fragments (relative to fragments of a reference peptide) were used to localize the modification site(s) for seven out of ten cases.Asp/iso-Asp and N-terminal modifications were not readily differentiated in these experiments, and the authors suggest that electron-based dissociation methods or additional IM experiments may be beneficial (83). Overall, this approach could speed up modification characterization in biopharmaceutical research.

Post-cIM activation takes advantage of the high-resolution IM separation of precursors, allowing unique fragmentation patterns to be linked to specific arrival-time distribution features and simplifying tandem MS (MS/MS) spectral interpretation. For instance, cIM sufficiently resolved a standard mixture of isomeric methylated ribonucleotide variants, and post-cIM CID produced diagnostic product ions that unambiguously confirmed the location of those modifications (84). Because methylation is a common post-transcriptional modification, cIM-CID was also applied to a biological mixture generated by exonuclease digestion of RNA from HeLa cells; the methyl-cytidine isobars and methyl-adenine isobars were identified, highlighting the ability of this method to handle complex epitranscriptomic analyses (84). Isobaric sets of mono- and disaccharides were also probed with this approach (85). Because these small saccharides have similar mobilities, a derivatization step further enhanced the IM separation (85). For derivatized disaccharides, positional isomers and functional derivatization isomers were unresolved with one cIM pass; three cIM passes resolved these isomers, but also resulted in wrap-around effects for some ions. Diagnostic product ions helped determine derivatization sites from resolved isomers, and their alignment with the arrival-time distributions for precursor helped to identify isomers for incompletely resolved features. Similarly, when considering a mixture of the sugar standards, those that were not fully resolved from one another in IM space could be distinguished by the cIM-CID fragment peaks.

**Figure 4:**
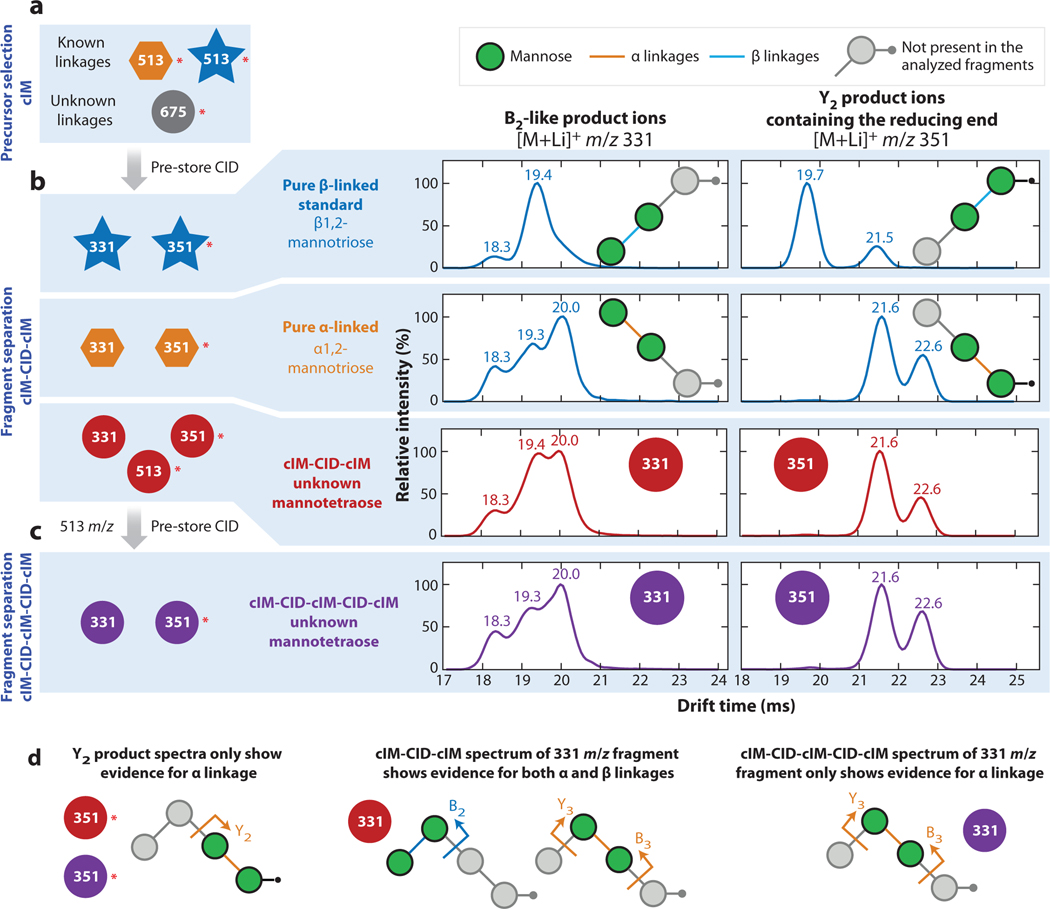
cIM-CID-cIM-CID-cIM scheme used to identify linkage anomericity of an intact, unknown mannotetraose (675 m/z). Red asterisks mark ions with the ^18^O-labeled reducing end. (*a*) In separate analyses, the intact mannotetraose (675 m/z; *circles*), a pure β-linked mannotriose standard (513 m/z; *stars*), and a previously characterized pure α-linked mannotriose (513 m/z; *hexagons*) underwent initial multipass cIM separations to isolate the major isoforms from contaminants and minor species. These precursor drift-time distributions are not shown. (*b*) The major isoforms were mobility selected, then fragmented on reinjection to the cIM region from the pre-store array (CID). All analytes produced fragments at 331 and 351 m/z; these underwent a 4-pass cIM separation. Their recorded drift-time distributions are shown on the right (cIM-CID-cIM). (*c*) The mannotetraose also produced a 513 m/z fragment (drift-time distribution not shown) that was mobility selected and fragmented again, producing ions at 331 and 351 m/z. After another 4-pass cIM separation, drift-time distributions were recorded (cIM-CID-cIM-CID-cIM); they are shown on the right. (*d*) Assignments of mannotetraose fragments (*arrows*) and linkages (*lines*) made based on mobility data and fitting. The cIM-CID-cIM spectrum of 331 m/z fragments contained a mixture of α and β linkages, arising from both ${\text{B}}_{2}$ fragments and isobaric ${\text{Y}}_{3}+{\text{B}}_{3}$ sequential fragmentation products. Abbreviations: CID, collision-induced dissociation; cIM, cyclic IM. Figure adapted with permission from Reference 86; copyright 2021 American Chemical Society.

Multiple stages of CID and cIM can be exploited to capture more information. For example, a selection of derivatized saccharides had asymmetric arrival-time distributions (85). To investigate the possibility of interconversion of the mannose derivatives, each feature was isolated in a cIM-CID-cIM experiment and activated on reinjection to the cIM region. The resulting arrival-time distributions were the same as those measured prior to CID, and no additional peaks were observed, indicating the presence of other mannose isomers instead. cIM improves analysis of more complex biological samples in this way as well. De novo structural determination of mannosides from crude biological media was performed with the inclusion of heavy oxygen labeling for MS/MS interpretation (86). cIM-CID elucidated the regioisomerism of the glycosidic bonds in the mannotriose and mannotetraose enzymatic products, whereas cIM-CID-cIM and cIM-CID-cIM-CID-cIM identified intrachain anomerism and verified that the anomericity was retained through multiple stages of fragmentation. This workflow revealed both α and β linkages in the mannotetraose, a novel finding for the product of a single enzyme. Figure 4 shows a representation of the cIM-CID-cIM-CID-cIM mannotetraose analysis. Work toward de novo sequencing of the human milk glycome has also benefited from high-resolution IM and multistage fragmentation (87). In addition, because tandem IM shows more capacity for isomer resolution than tandem MS alone, a molecular networking strategy, similar to those used for MS/MS data mining, was employed to better sort oligosaccharides for glycomics (88). cIM-CID-cIM was applied to a set of 33 penta- and hexasaccharides, and the resulting IM and MS data were used to create two networks. The resulting clusters and subclusters were compared, and the tandem IM network could be justified according to regioisomerism, epimerism, and anomerism, clearly sorting with sensitivity to isomeric structural features (88). These novel ion manipulation strategies have been applied to the analysis of other carbohydrates (89, 90) as well as proteins (91–95), synthetic polymers (96), and crude oil (97, 98).

Many of the aforementioned studies focused on differentiating elementary units and small molecules toward the analysis of larger biomolecules. Enzymatic digestion-based protocols have also been used in combination with the high resolution and multifunctionality of the cIM system to accomplish proteomic and glycomic goals. For example, expanding into larger polysaccharide analyses, red algal cell walls were digested, resulting in octomeric oligoporphyrans, and multistage cIM with post-cIM fragmentation not only sequenced the porphyrans but also localized the methyl ether and sulfate groups (89).In a bottom-up proteomics approach,an IgG4 mAb with two noncanonical cysteine residues was tryptically digested and subjected to multipass IM (99). The resulting disulfide-linked dipeptides were unambiguously identified by comparing high-resolution arrival-time distribution profiles with potential peptide isomers; the number and location of the linkages were determined with only high-resolution IM (99). Size-exclusion chromatography and high-resolution IM were used to confirm the presence of two coexisting conformers of an anti-HIV mAb (91). High-resolution cIM of the isomerized subunit resolved these two isomers using four passes, whereas trapping in the pre-store region demonstrated that isomer interconversion was minimal (91).

The cIM system is also being applied to the structural analysis of intact proteins. Although experiments that use longer separation lengths can yield higher resolving powers (Figure 1),those experiments also increase the time available for structural isomerization. Analysis of native-like ions of cytochrome c (12-kDa monomer), β-lactoglobulin (16-kDa monomer), and concanavalin A (51-kDa dimer or 102-kDa tetramer) suggest that the mobilities of the low-mass protein ions exhibit subtle changes over those timescales, but those of the high-mass protein ions do not (100). Follow-on studies highlighted the unique capabilities of the cIM system to probe phenomena such as sequential unfolding, irreversible versus reversible unfolding, and interconversion during the separation (92).Additionally,to investigate the conformational dynamics of even larger native-like and aggregated biomolecules with cIM, the transmission of intact proteins (1.5 MDa) and oligonucleotides (63 kDa) was demonstrated (101).With the recent integration of complementary dissociation techniques, including electron-capture dissociation (90, 94) and surface-induced dissociation (95), the cIM system offers many exciting possibilities for hybrid, top-down MS experiments.

## TRAPPED ION MOBILITY SPECTROMETRY

Thus far, we have only discussed implementations of IM whose performance depends on the distance that ions traverse in the laboratory frame of reference, through single or multiple passes. In TIMS, a gas flow propels ions forward against a repulsive gradient of E (102). Ions reach an equilibrium position that corresponds to where the electrostatic force equals the counteracting drag force (103). As the electric field gradient is decreased, ions of lower K elute from the device first as the drag force overcomes the now-reduced electrostatic force. High-resolution separations can be achieved by tuning the scanning speed of the gradient (21); note that under a given set of conditions lower-K ions will generally exhibit a larger value of ${R_{p}}^{\text{Ω}}$. TIMS separations have achieved an ${R_{p}}^{\text{Ω}}$ of ~400 in separating singly charged polybrominated diphenyl ether metabolites (104), whereas an ${R_{p}}^{\text{Ω}}$ of 295 was observed for an elongated conformer of 7+ ubiquitin (105). Beyond tunable resolving power, TIMS boasts high sensitivity owing to both the radial confinement and higher capacities for ion accumulation, as well as the ability to measure Ω values in agreement with values determined using drift-tube IM (37).These features, introduced by Bruker Co., make TIMS an accessible and potent IM platform, as evidenced by the number of recent publications ranging in application from lipidomics (106–109), glycomics (110, 111), proteomics (112–116), and beyond. One particularly noteworthy application of TIMS is its synchronization with MS/MS to increase the sensitivity of both data-dependent (106) and data-independent (117) omics workflows. Previous reviews provide a more comprehensive summary of TIMS operation (28) and its various applications to biomolecules (37).

Other next-generation innovations using TIMS have focused on adding orthogonality. In one study, TIMS was combined with surface-induced dissociation (SID), which enabled the charge state–dependent dissociation of protein complexes and the conformation-dependent dissociation of isobaric peptides (118). In analyzing SID products, observed charge-state distributions and retention of bound ligands indicated that TIMS can probe native-like structures with properly tuned parameters (118). A TIMS-electron capture dissociation workflow was used to characterize the post-translational modifications of histone tails (119). TIMS was used to address analytical difficulties owing to the isobaric and isomeric nature of these post-translational modifications, resolving different conformations such that subsequent electron capture dissociation revealed the location of the post-translational modifications (119). TIMS was also combined with ultraviolet photodissociation, which was used to characterize isobaric and isomeric species at ${R_{p}}^{\text{Ω}}$ values >100 (120). Another implementation of the TIMS platform coupled with electronic excitation dissociation revealed isomeric-specific glycan fragments that were consistent across multiple observed conformations for individual glycans (121). Taken as a whole, these studies illustrate the potential for TIMS to address shortcomings in top-down proteomics, lipidomics, and glycomics studies hindered by isomeric and isobaric species. Furthermore, the ability to vary the scanning speed enables the facile synchronization of TIMS separations with high-resolution, albeit lower duty cycle mass analyzers such as Fourier-transform ion cyclotron resonance (122).

The TIMS technology has been further advanced on prototype instruments in research laboratories. Fernandez-Lima and coworkers (123) introduced a TIMS cell with an extended mass range enabled by convex electrodes that increase the penetration of the pseudopotential well. Ω values were obtained for a wide range of native-like proteins and protein complexes, ranging from ubiquitin to GroEL, and those values were consistent with those from drift-tube IM (123). Since its introduction, this system has been used to elucidate the binding dynamics of an intrinsically disordered DNA-binding protein; results from collision-induced unfolding experiments exhibited evidence for increased protein stability upon DNA binding (124).

**Figure 5:**
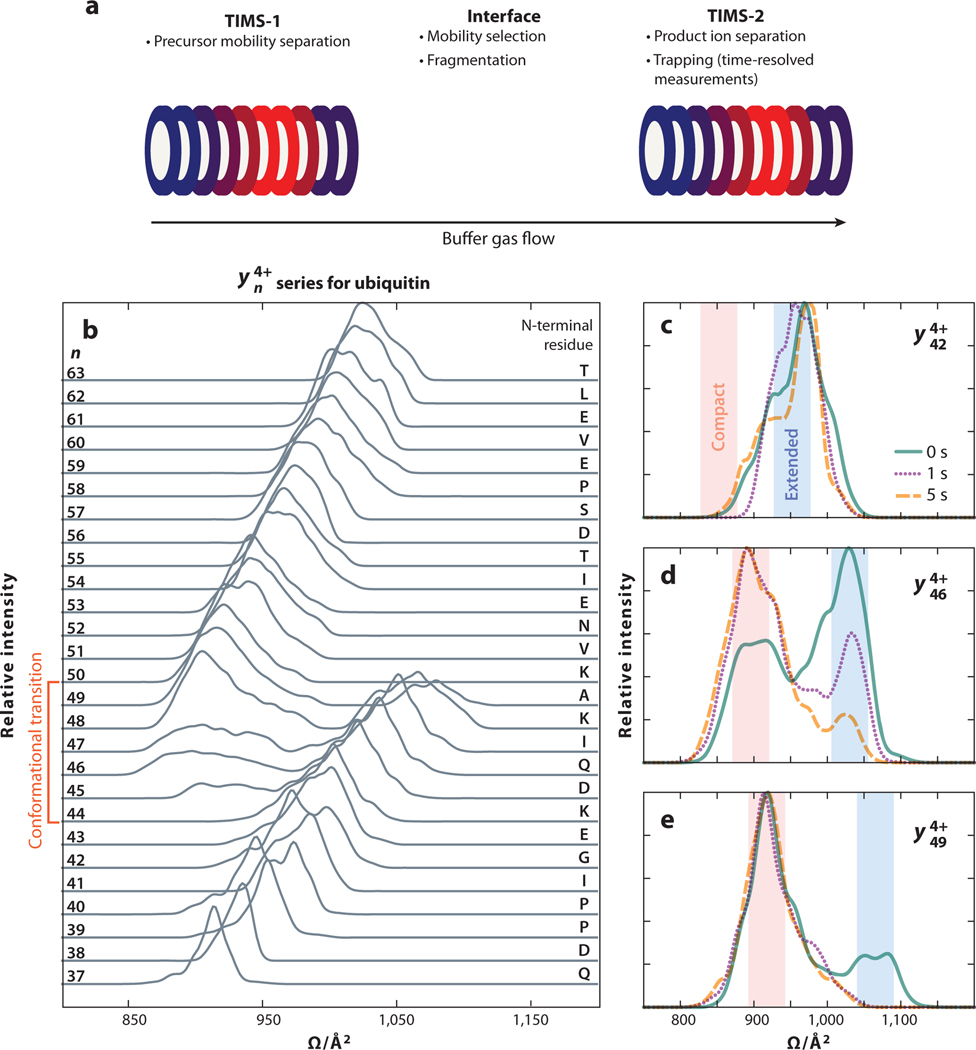
(*a*) Schematic of the tandem TIMS implementation reproduced using the graphics from Figure 1; the system used for these experiments has been reported (127). (*b*) IM spectra of the ${y_{n}}^{4+}$ fragment series of ubiquitin. At ${y_{44}}^{4+}$, compaction is observed as the Ω values of the fragment ions decrease with increasing number of residues. Panels *c*, *d*, and *e* show the time-dependent IM distributions of selected ions of the fragment series. Whereas ${y_{42}}^{4+}$ and ${y_{49}}^{4+}$ remain primarily in extended and compact conformations respectively, ${y_{46}}^{4+}$ undergoes a time-dependent compaction that is evidence for gas-phase folding. Abbreviations: IM, ion mobility; TIMS, trapped IM spectrometry. Panels *b*–*e* adapted with permission from Reference 127; copyright 2022 American Chemical Society.

Bleiholder and coworkers (125) introduced TIMS-TIMS, or tandem TIMS; implementations and early applications of this technology were reviewed recently (126).Briefly,the first implementation consisted of two prototype TIMS devices, which are referred to as TIMS-1 and TIMS-2, interfaced coaxially using ion apertures capable of collisional activation (125). One consideration for the coaxial device involves defining the gas flow between the devices; pressure differentials between TIMS-1 and TIMS-2 result in a trade-off between resolution and ion activation (126). More recently, the coaxial device was used to analyze the gas-phase structures of top-down protein fragments (127) (Figure 5). Following mobility selection and fragmentation, Ω measurements in TIMS-2 revealed that top-down fragments exhibited structural heterogeneity and multiple conformations. Notably, the Ω distributions of the ${y_{n}}^{4+}$ fragment series of ubiquitin show a compaction event upon the incorporation of a basic residue (127) (Figure5b). Time-resolved, TIMS-2 measurements of the ${y_{n}}^{4+}$ fragment series provide evidence for the formation of metastable conformations; ions that exhibited both compact and extended conformations underwent folding on the seconds timescale (127) (Figure 5c–e). These experiments illustrate the potential of multidimensional IM experiments to inform the relationship between precursor and product ion structure in top-down proteomics. A more recent implementation interfaces two commercial TIMS devices orthogonally, separated by an ion trap to enable ultraviolet photodissociation (128). Ion trap conditions were sufficiently soft to maintain native-like structures, which were then fragmented prior to TIMS-2 separations that enabled increased fragment identification (128). Despite limitations of available activation methods accessible at TIMS-TIMS pressure regimes (126), this approach is poised to offer unique insights into structural proteomics.

## CONCLUSIONS AND FUTURE PERSPECTIVES

Sustained innovation continues to redefine the performance of IM-enabled experiments (Figure 1). These advances have already had great impacts on the ability of MS-based measurements to analyze isomeric and isobaric species, e.g., as evidenced by the extensive application of SLIM (Figures 2 and 3), cIM (Figure 4), and TIMS to glycans. Multidimensional experiments that activate ions between dimensions of IM increase information content by enabling conformer-dependent unfolding and fragmentation.This feature has been elegantly implemented on multiple platforms, including the SLIM/cryo-IR (Figure 3), cIM (Figure 4), and TIMS-TIMS (Figure 5) systems. Many initial applications of the next-generation technologies summarized in Figure 1 have focused on glycomics, lipidomics, and peptidomics. There has been a long-standing interest in using IM-MS as a high-throughput replacement for LC-MS. The applications described here demonstrate the effectiveness of IM to resolve the components of mixtures that had previously required LC and that had not been successfully separated using LC. We note that LC-MS methods benefit from the peak capacity of LC separations, as well as the ability of LC systems to manage samples and deliver eluent that is amenable to electrospray ionization (e.g., by desalting samples). For routine, high-throughput measurements, we propose that IM-enabled methods will benefit greatly from using LC, IM, and MS in tandem, i.e., using fast, minimal LC to manage and prepare samples for ionization and then using next-generation IM experiments to add the necessary level of selectivity.

As expected, new innovations tend to reveal new challenges. For example, collisional activation may be insufficient to fragment many intact protein ions. Complementary methods of activation combined with high-resolution IM may be especially useful for differentiating the components that contribute to the congestion that is characteristic of top-down mass spectra. Instrument geometries that support multiple passes, including the cIM and some implementations of TW-SLIM, enable methods that increase the pathlength to achieve the required resolution for an application. However, multiple passes can also result in ion wrap-around, which can hinder data analysis or require the analysis to focus on progressively narrower mobility ranges while discarding other ions as the separation length is increased. IM separations using long geometries and/or multiple passes take longer to perform, which makes it more challenging to use the entire flux produced by the ion source and increases the likelihood of structural isomerization and ion chemistry during experiments. More generally, multidimensional experiments often yield complex data that resist simple analysis workflows. Nevertheless, we suspect that researchers will continue to innovate, that these and other challenges will be addressed, and that these next-generation IM technologies will fulfill even greater roles in biomolecular analysis in the years ahead.
